# Gold Nanoparticle-Catalyzed Environmentally Benign Deoxygenation of Epoxides to Alkenes

**DOI:** 10.3390/molecules16108209

**Published:** 2011-09-28

**Authors:** Akifumi Noujima, Takato Mitsudome, Tomoo Mizugaki, Koichiro Jitsukawa, Kiyotomi Kaneda

**Affiliations:** 1 Department of Materials Engineering Science, Graduate School of Engineering Science, Osaka University, 1-3, Machikaneyama, Toyonaka, Osaka 560-8531, Japan; 2 Research Center for Solar Energy Chemistry Osaka University, 1-3, Machikaneyama, Toyonaka, Osaka 560-8531, Japan

**Keywords:** gold nanoparticle, hydrotalcite, deoxygenation, epoxide, alkene

## Abstract

We have developed a highly efficient and green catalytic deoxygenation of epoxides to alkenes using gold nanoparticles (NPs) supported on hydrotalcite [HT: Mg_6_Al_2_CO_3_(OH)_16_] (Au/HT) with alcohols, CO/H_2_O or H_2_ as the reducing reagent. Various epoxides were selectively converted to the corresponding alkenes. Among the novel metal NPs on HT, Au/HT was found to exhibit outstanding catalytic activity for the deoxygenation reaction. Moreover, Au/HT can be separated from the reaction mixture and reused with retention of its catalytic activity and selectivity. The high catalytic performance of Au/HT was attributed to the selective formation of Au-hydride species by the cooperative effect between Au NPs and HT.

## 1. Introduction

**Scheme 1:**
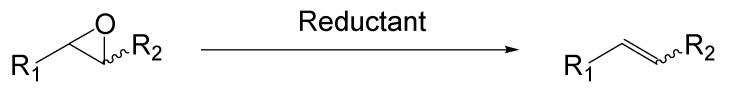
Deoxygenation of epoxides to the corresponding alkenes.

The chemoselective reduction of organic compounds is one of the most fundamental reactions in organic chemistry. Among these reductive reactions, the deoxygenation of epoxides into alkenes (Scheme 1) has attracted much attention in both organic synthesis and biological chemistry where, for example, it occurs in the protection-deprotection cycle of carbon-carbon double bonds [1,2,3] and in the production of vitamin K in the human body [4,5].

Generally, stoichiometric deoxygenations of epoxides have been conducted using expensive and/or toxic reagents such as low valent metals [6,7,8,9,10,11,12,13], iodides [14,15,16,17], phosphines [18,19,20], and silane compounds [21,22,23]. These reagents are often employed in excess, resulting in the production of large amounts of waste. To date, catalytic deoxygenations using Re complexes with triphenylphosphine [24,25,26], a Fe complex with NaBH_4_ [27], and a Co complex with Na [28] have been reported, but these homogeneous catalytic systems have suffered from tedious work-up procedures, air- and moisture-sensitive reaction conditions, low atom efficiency and low catalytic activities. Therefore, the development of highly efficient and environmentally benign catalytic deoxygenation of epoxides is still a challenging issue.

Gold nanoparticles (Au NPs) have received much attention in the catalysis field due to their unique and high oxidation ability in various reactions such as oxidation of CO [29] and alcohols [30,31], and epoxidation of alkenes [32,33]. On the other hand, the reduction ability of the Au NP catalysts has not yet been widely studied despite their remarkable activities [34,35]. We have focused on exploring the novel catalytic reactions of Au NPs, and found that supported Au NPs showed unprecedented reduction activities in the deoxygenation of epoxides [36,37,38], *N*-oxides, sulfoxides, and amides [39].

**Scheme 2:**
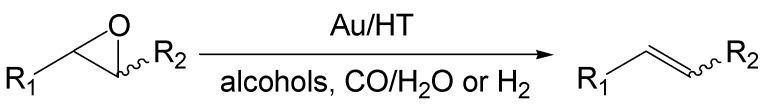
Deoxygenation of epoxides catalyzed by Au/HT.

In this account, we review our recent progress on the unique catalysis of Au NPs supported on hydrotalcite [HT: Mg_6_Al_2_CO_3_(OH)_16_] (Au/HT) for deoxygenation of epoxides to the corresponding alkenes using alcohols [36] or CO/H_2_O [37] as reductant (Scheme 2). A wide range of epoxides were deoxygenated to the corresponding alkenes with over 99% selectivities. The C=C bonds of the products were not hydrogenated at all. After the reaction, the solid Au/HT catalyst could be easily separated and reused without loss of its activity or selectivity. Furthermore, Au/HT was successfully applicable to an ideal deoxygenation, *i.e.*, the H_2_-mediated deoxygenation of epoxides where only water is formed as a by-product [38].

## 2. Results and Discussion

### 2.1. Characterization of Au/HT

**Figure 1:**
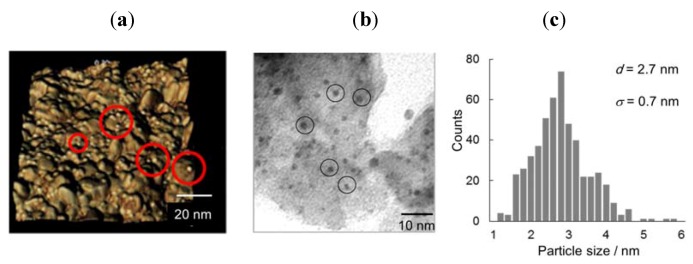
AFM (**a**); TEM (**b**) and size distribution (**c**) of Au NPs on HT.

Au/HT was prepared by the deposition precipitation method (see Experimental). From atomic force microscopy (AFM) and transmission electron microscopy (TEM) analyses, the mean diameter (*d*) of Au NPs on the surface of HT was 2.7 nm with a standard deviation (*σ*) of 0.7 nm (Figure 1).

### 2.2. Deoxygenation of Epoxides to Alkenes

#### 2.2.1. Au/HT-Catalyzed Deoxygenation of Epoxides Using Alcohols as a Reductant

**Scheme 3:**
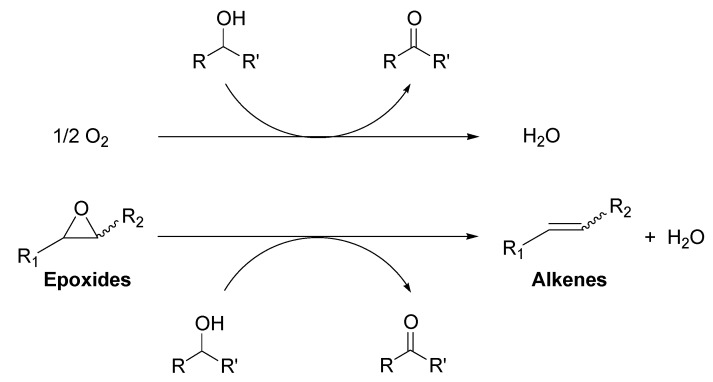
The oxidation of alcohols using O_2_ *vs.* deoxygenation of epoxides using alcohols.

Recently, we have found that Au/HT could catalyze the highly efficient aerobic oxidation of alcohols [40] and the lactonization of diols [41]. These results allowed us to predict that if epoxides could be employed as hydrogen acceptors in place of molecular oxygen under the alcohol oxidation conditions, a green catalytic deoxygenation of epoxides with alcohols could be developed (Scheme 3) [36].

To demonstrate the above hypothesis, we carried out the deoxygenation of *trans*-stilbene oxide (**1**) using Au/HT with 2-propanol in toluene as the solvent at 110 °C under Ar atmosphere for 4 h. **1** was successfully deoxygenated to give the corresponding alkene *trans*-stilbene (**2**) in quantitative yield. Notably, no by-products such as 1,2-diphenylethanol or 1,2-diphenylethane resulting from the hydrogenation of **1** or **2** were formed (Table 1, Entry 1). During the deoxygenation of **1**, the amounts of acetone and water generated were almost equivalent to that of **2**. Among the alcohols tested, 1-phenylethanol and benzyl alcohol could also function as reductants (Entries 2 and 3), while the use of an aliphatic primary alcohol such as 1-octanol resulted in lower yield (Entry 4). Next, the effects of inorganic supports of Au NPs were investigated. Au NPs on basic supports of Al_2_O_3_ and MgO afforded good to moderate yields of **2** (Entries 5 and 6), whereas non-basic supports like TiO_2_ and SiO_2_ were not effective (Entries 7 and 8). Other Au compounds like HAuCl_4_, Au_2_O_3_ and bulk Au metal did not promote the deoxygenation (Entries 9–11). These results indicate that the combination of Au NPs and a basic support is necessary to achieve the high catalytic activity for the deoxygenation.

**Table 1:** Deoxygenation of *trans*-stilbene oxide ^a^. 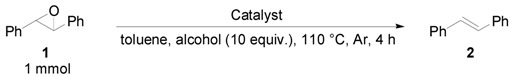

Entry	Catalyst	Alcohol	Conv. ^b^ (%)	Sel. ^b^ (%)
1	Au/HT	2-propanol	99	>99	
2	Au/HT	1-phenylethanol	99	>99	
3	Au/HT	benzyl alcohol	91	>99	
4	Au/HT	1-octanol	37	>99	
5	Au/Al_2_O_3_	2-propanol	60	>99	
6	Au/MgO	2-propanol	43	>99	
7	Au/TiO_2_	2-propanol	19	>99	
8	Au/SiO_2_	2-propanol	5	>99	
9	HAuCl_4_	2-propanol	<1	-	
10	Au_2_O_3_	2-propanol	<1	-	
11	bulk Au metal	2-propanol	0	-	

^a^ Au cat. (0.45 mol%), toluene (5 mL), alcohol (10 mmol); ^b^ Determined by HPLC using an internal standard technique.

**Figure 2:**
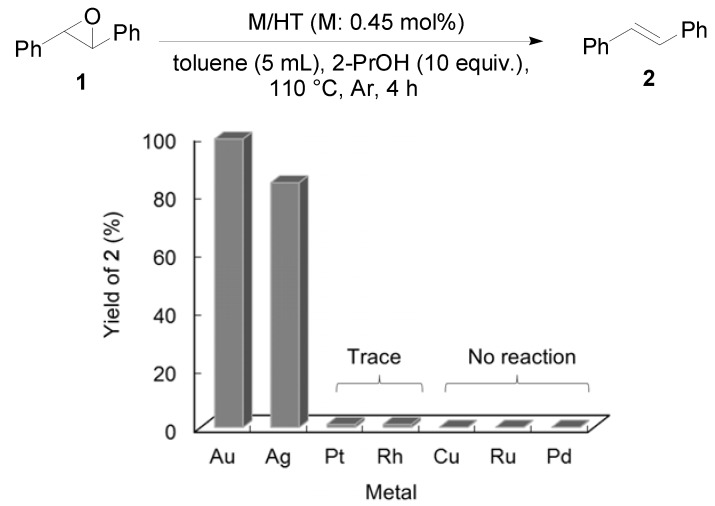
Deoxygenation of *trans*-stilbene oxide using HT-supported various metal NPs.

Other metal NPs on HT were examined for the deoxygenation of **1** (Figure 2). Among the catalysts tested, Ag/HT also showed excellent catalytic activity for deoxygenation, while other metal NPs did not function as catalysts.

To investigate the possibility of the leaching of active metal species from Au/HT into the reaction mixtures, Au/HT was filtered from the reaction mixture at 50% conversion of 1, and treatment of the filtrate with additional stirring under similar conditions did not give any product. Furthermore, inductively coupled plasma atomic emission spectral (ICP-AES) analysis revealed no Au species in the filtrates (detection limit: 0.1 ppm). These results clearly proved that no leaching occurred and the deoxygenation proceeded on the Au NPs on HT.

The outstanding catalytic activity of Au NPs encouraged us to investigate the scope of epoxides in the deoxygenation (Table 2). Various epoxides were efficiently converted into the corresponding alkenes with over 99% selectivity. Both aromatic and aliphatic epoxides could be deoxygenated. Epoxides having ether and hydroxyl groups were also successfully employed as substrates (Entries 9 and 16). Notably, the reducible C=O bonds of epoxyketones were tolerated in the deoxygenation (Entries 14 and 15). *cis*-Stilbene oxide and *cis*-2,3-epoxyoctane gave (*Z*)/(*E*)-alkene stereoisomers. The selectivities for *cis*-alkenes were 60% and 50%, respectively (Entries 4 and 13).

**Table 2:** Deoxygenation of various epoxides using Au/HT ^a^ . 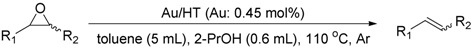

Entry	Substrate	Product	Time (h)	Conv. ^b^ (%)	Yield ^b^ (%)
1	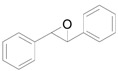	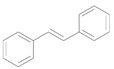	4	99	99
2 ^c^			4	99	99
3 ^d^			4	97	97
4	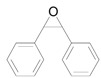	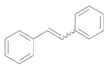	4	99	97 ( *E/Z*) = 2/3
5	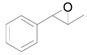	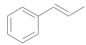	4	98	98
6 ^e^	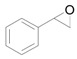	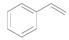	6	>99	98
7	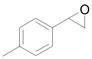	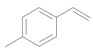	6	96	92
8	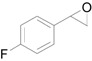	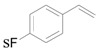	6	94	91
9	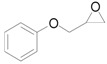	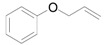	6	93	90
10	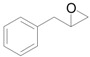	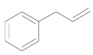	24	89	89
11	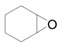	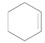	24	87	87
12 ^e^	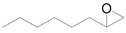	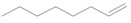	4	>99	97
13 ^f^	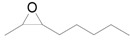	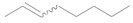	12	81	77 (E/Z) = 1/1
14 ^f^	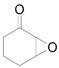	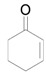	24	88	85
15 ^f^	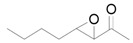	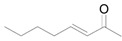	24	99	94
16 ^f^	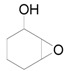	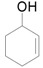	6	93	91
17	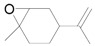	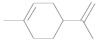	24	72	68

^a^ Reaction conditions: Au/HT (0.1 g), substrate (1 mmol), Ar; ^b^ Determined by GC or HPLC using an internal standard technique; ^c^ Reuse 1; ^d^ Reuse 2; ^e^ 80 °C; ^f^ Substrate (0.3 mmol), catalyst (0.2 g).

**Figure 3:**
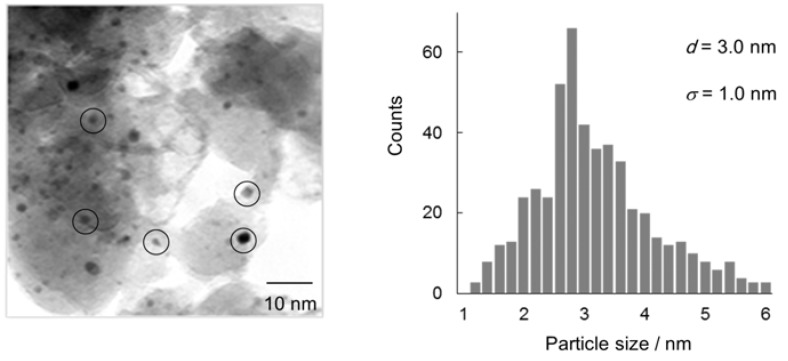
TEM image and size distribution of Au NPs of Au/HT after reuse experiments.

After the deoxygenation of **1**, the solid Au/HT catalyst could be easily separated from the reaction mixtures and reused with retention of its performance (Entries 2 and 3). TEM images showed that the Au NPs on HT after reuse were similar to fresh Au/HT in average diameter and size distribution and no aggregation of the used Au NPs was observed (Figure 3).

Atomic-scale analysis using Au L-edge EXAFS of Au/HT showed that the intensity of the FT peak derived from the Au-Au shell at 2.8 Å was not changed, supporting the observation that the Au NPs after reuse were of the same size as the originals. These results are consistent with the high durability of Au/HT in the recycling experiments.

**Scheme 4:**
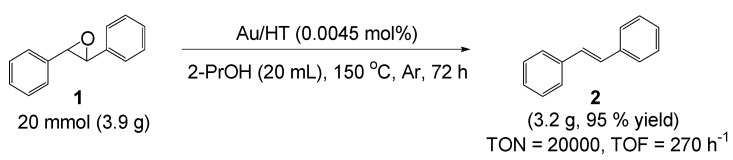
Preparative deoxygenation of *trans*-stilbene oxide using Au/HT.

Au/HT was also applicable in a preparative scale reaction (Scheme 4). Thus, the deoxygenation of 20 mmol of **1** successfully proceeded to afford **2** with 95% isolated yield in 2-propanol as solvent after 72 h, where the TON and TOF reached 20,000 and 270 h^−1^, respectively. These values are three orders of magnitude greater than those of previously reported catalytic systems such as Tp’ReO_3_-PPh_3_ (TON = 19) [24], polystyrene-supported Re-PPh_3 _(TON = 18, TOF = 12 h^−1^) [26], CH_3_ReO_3_-PPh_3_ (TON = 8, TOF = 0.4) [25], [Fe_4_S_4_(SC_6_H_5_)_4_]^3−^-NaBH_4_ (TON = 4, TOF = 1 h^−1^) [27], and Co complex-Na (Hg) (TON = 5, TOF = 0.8 h^−1^) [28].

**Scheme 5:**
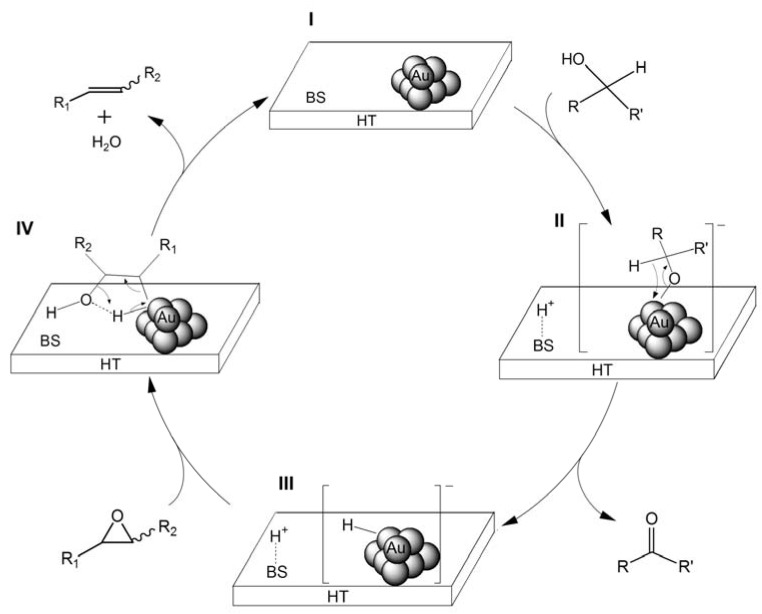
A plausible reaction mechanism for the Au/HT-catalyzed deoxygenation of an epoxide through the cooperation of the Au NPs with basic sites (BS) of HT.

In separate experiments, the use of *d*-benzhydrol [C_6_H_5_CD(OD)C_6_H_5_] as a reductant for the deoxygenation of **1** afforded **2** and D_2_O with all hydrogen atoms in the alkene product retained. From both these results and the positive effect of basic supports as shown in Table 1 (Entries 1, 5 and 6), we propose the following mechanism as shown in Scheme 5.

A basic site (denoted as BS) of HT abstracts the H^+^ from the hydroxyl group of the alcohol to promote the formation of an Au-alcoholate species which subsequently forms an Au-hydride species and the corresponding carbonyl compound through β-hydride elimination [42]. The Au-hydride species and H^+^ attack an epoxide, providing an alkene and H_2_O. The distinguished deoxygenation activities of Au NPs from those of other metal NPs should be attributed to the reactivity toward an epoxides (III→I) because the other metal NPs of Cu, Ru, Pd and Rh, which can form metal-hydride species from the reaction with the alcohol (I→III), do not deoxygenate the epoxide.

#### 2.2.2. Deoxygenation of Epoxides with CO/H2O

**Scheme 6:**
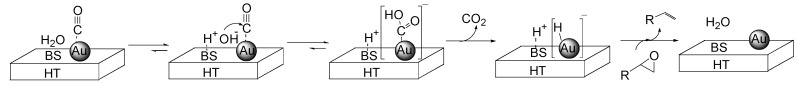
Concerted effects between HT and Au NPs for the formation of Au-H species (BS represents base site of HT).

We have previously reported that Rh carbonyl species can deoxygenate nitro compounds in the presence of amines as bases under water-gas shift reaction conditions (CO + H_2_O → CO_2_ + H_2_) [43]. In this reaction, CO and H_2_O react with the Rh carbonyl species and an amine to form a Rh-hydride species that is active for the reduction of nitro compounds. The formation of the Rh-hydride species in cooperation with amines under water-gas shift reaction conditions inspired us to develop an alternative catalytic deoxygenation system using Au/HT. Namely, we proposed that an active Au-hydride species for the deoxygenation of epoxides can be formed through the cooperative effect of HT as a base under water-gas shift reaction conditions (Scheme 6). Thus, the attack of H_2_O on the basic sites of HT to CO adsorbed on Au NPs generates [Au-COOH]^−^, followed by the elimination of CO_2_ to give the Au-hydride species and H^+^, which then act in concert to deoxygenate epoxides to alkenes [37].

Based on the above hypothesis, we carried out the deoxygenation of styrene oxide (**1a**) under water-gas shift reaction conditions in the presence of Au/HT. Styrene (**2a**) was quantitatively obtained as the sole product under atmospheric pressure CO in water at room temperature (Table 3, Entry 1). Various epoxides tested in the Au/HT-2-propanol system were also reactive under water-gas shift conditions (Table 4). Compared with the Au/HT-2-propanol system, this Au/HT-CO/H_2_O system can promote the deoxygenation of epoxides under mild and convenient reaction conditions, e.g., at room temperature, 1 atm of CO, and in the absence of organic solvents.

**Table 3:** Deoxygenation of styrene oxide using CO/H_2_O ^a^. 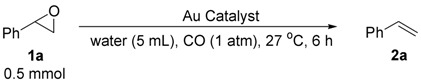

Entry	Catalyst	Yield ^b^ (%)	Sel. ^b^ (%)
1	Au/HT	>99	>99
2 ^c^	Au/HT	>99	>99
3 ^d^	Au/HT	97	>99
4	Au/Al_2_O_3_	79	>99
5	Au/MgO	36	>99
6	Au/TiO_2_	18	>99
7 ^e^	Au/TiO_2_ + Na_2_CO_3_	57	>99
8	Au/SiO_2_	3	>99

^a^ Reaction conditions: catalyst (M: 0.9 mol%); water; ^b^ Determined by GC using an internal standard technique; ^c^ Reuse 1; ^d^ Reuse 2; ^e^ Na_2_CO_3_ (1.5 mmol) was added.

**Table 4:** Deoxygenation of various epoxides with CO/H_2_O using Au/HT ^a^. 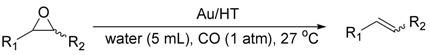

Entry	Substrate	Product	Time (h)	Yield ^b^ (%)	Sel. ^b^ (%)
1	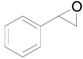	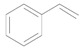	6	>99 (94)	>99
2	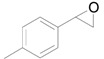	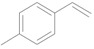	12	90 (85)	>99
3	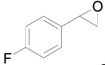	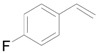	12	99 (94)	>99
4	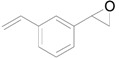	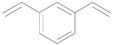	16	90 (85)	>99
5	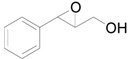	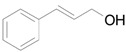	16	96 (90)	96
6	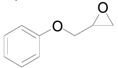	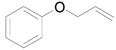	24	90 (83)	>99
7	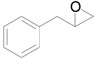	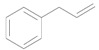	24	75 (69)	>99

^a^ Reaction conditions: Substrate (0.5 mmol), Au/HT (0.1 g, Au: 0.9 mol%); ^b^ Determined by GC using an internal standard technique. Values in parentheses are isolated yields; ^c^ Cinnamaldehyde was formed as a byproduct.

**Scheme 7:**
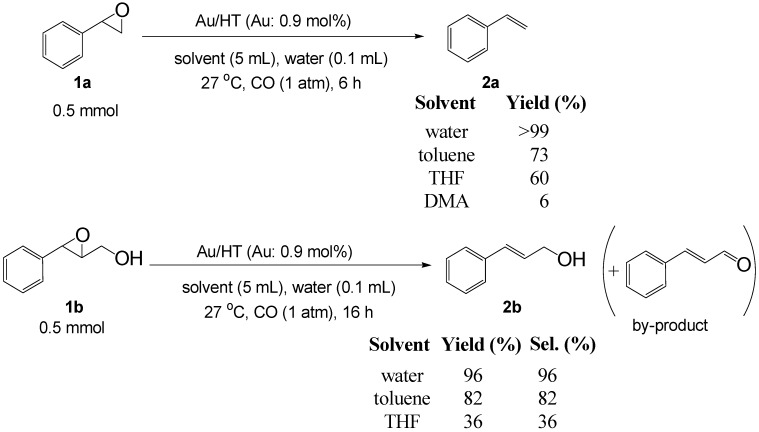
Solvent effect on the deoxygenation of epoxides.

The solvent effect on the deoxygenation is shown in Scheme 7. Notably, water was found to provide the highest yield among all the solvents tested despite the water-insoluble nature of **1a**. In the case of the epoxy alcohol 2,3-epoxy-3-phenyl-1-propanol (**1b**), the highest yield and selectivity of cinnamyl alcohol (**2b**) were obtained in water. After the deoxygenation of **1a**, **2a** was easily extracted from the reaction mixture by *n*-hexane and the recovered aqueous phase containing solid Au/HT could be recycled with no decrease in catalytic activity (Table 3, Entries 2 and 3).

**Figure 4:**
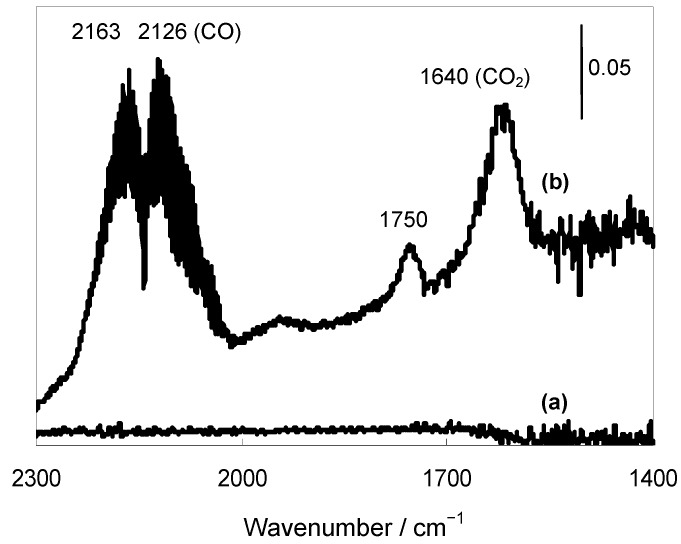
FTIR spectra of (a) Au/HT and (b) after CO/H_2_O adsorption at 298 K.

To gain more insight into the deoxygenation under the water-gas shift conditions, the following control experments were carried out. When the reaction was conducted in the absence of **1a** under otherwise identical conditions, H_2 _was not detected. The use of D_2_O in place of H_2_O significantly affected the reaction rate for the deoxygenation of **1a** with a *k_H_/k_D_* value of 3.9. These results rule out the participation of H_2_ in the Au/HT-catalyzed deoxygenation reaction, while indicating that not only CO functions as a sole reductant, but also water takes part in the deoxygenation. An additional experiment using *trans*-2-octenal in place of **1a** revealed that chemoselective reduction occurred to give *trans*-2-octen-1-ol as the sole product while retaining the C=C double bond of the starting material. From the above results, we believe that an active Au-hydride species is generated in situ from the reaction of H_2_O with CO during the deoxygenation of epoxides. According to the proposed reaction mechanism (Scheme 6), a basic site of HT facilitates the formation of the Au-hydride species through the nucleophilic attack of OH^−^ on the Au-CO species followed by a decarboxylation, which is well evidenced by the positive effect of the additional base of Na_2_CO_3_ to the Au NPs on the non-basic material of TiO_2_ (Table 3, Entry 6 *vs.* 7). Finally, to confirm the generation of the Au-H species, Fourier transform infrared (FT-IR) studies of Au/HT were conducted in the presence of CO and H_2_O. When Au/HT was treated with CO and H_2_O vapor at 298 K, a new band attributed to the generation of Au-H species was detected around 1750 cm^−1^ (Figure 4). Next, this treated Au/HT was exposed to the vapor of **1a**, and the band attributed to the Au-H species gradually disappeared. The detection of the Au-H species agreed with recent IR and theoretical studies on Au–H species that predicted a band around 1800 cm^−1^ [44,45]. These above control experiments are consistent with the proposed reaction mechanism as shown in Scheme 6. The heterolytic H^+^ and Au-hydride species generated in situ on Au/HT deoxygenate the epoxide to form the corresponding alkene and water.

After the publication of our Au/HT-CO/H_2_O catalyst system, Cao *et al.* reported a deoxygenation method using Au/TiO_2_-VS (very small gold NPs on TiO_2_) under water-gas shift reaction conditions [46]. Au/TiO_2_-VS showed high catalytic activity (TON = 9,600, TOF = 400 h^−1^) in the deoxygenation of styrene oxide in the mixed solvent of acetone with H_2_O under a high pressure CO atmosphere (10 atm).

#### 2.2.3. Selective Deoxygenation of Epoxides Using Molecular Hydrogen

The ideal green methodology for the catalytic deoxygenation of epoxides is to utilize molecular hydrogen (H_2_) as a reductant due to the formation of non-toxic water as a by-product. However, the use of H_2_ often causes non-selective reduction of epoxides to yield alcohols and alkanes as byproducts through hydrogenation of the epoxides and overhydrogenation of the desired alkenes, respectively. Although there are a few successful reports on the selective deoxygenation of epoxides using H_2_, high selectivity for alkenes is restricted to low conversion levels [46] or a limited range of substrates [47]. Therefore, the development of an efficient catalytic system for the selective deoxygenation of epoxides to the corresponding alkenes using H_2_ is a challenging task.

With supported Au NPs in hand, the deoxygenation conditions were optimized [38]. When the deoxygenation of **1a** using Au/HT was carried out at 80 °C under 1 atm of H_2_ for 6 h, **1a **was converted to **2a** in 97% yield accompanied by the formation of ethylbenzene (**3a**) as a byproduct through the hydrogenation of the desired product **2a** (Table 5, Entry 1). Next, Au NPs on other supports were investigated in the deoxygenation of **1a** under similar reaction conditions. Au/CeO_2_ and Au/Al_2_O_3_ had lower selectivities for **2a**, which caused hydrogenation of **2a** (Entries 4 and 5) [48]. Interestingly, Au/TiO_2_ gave **2a** with over 99% selectivity, though the conversion of **1a** was much lower than that of Au/HT (Entry 6). Au/SiO_2_ did not exhibit any catalytic activity toward this reaction (Entry 7). Remarkably, when the reaction was carried out at 60 °C for 8 h, Au/HT produced **2a** in over 99% yield without formation of any side products (Entry 2). Moreover, the C=C bond of **2a** was completely intact when the reaction time was prolonged (Entry 3). The Au NP catalysis exhibited different activity from other metal NPs. Ag/HT, Rh/HT, Ru/HT, and Cu/HT did not function as catalysts for this reaction (Entries 10-13). On the other hand, Pd/HT and Pt/HT afforded undesired **4a** with over 99% selectivity through the hydrogenation of **1a** (Entries 8 and 9).

**Table 5:** Deoxygenation of styrene oxide using H_2_^ a^. 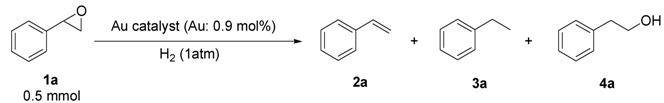

Entry	Catalyst	Temp. (°C)	Time (h)	Conv. ^b^ (%)	Sel. for 2a ^b^ (%)	Sel. for 3a ^b^ (%)	Sel. for 4a ^b^ (%)
1	Au/HT	80	6	97	97	3	0
2	Au/HT	60	8	>99	>99	0	0
3	Au/HT	60	24	>99	>99	0	0
4	Au/CeO_2_	80	6	64	81	19	0
5	Au/Al_2_O_3_	80	6	82	36	64	0
6	Au/TiO_2_	80	6	26	>99	<1	0
7	Au/SiO_2_	80	6	<1	-	-	-
8	Pd/HT	80	6	>99	0	0	>99
9	Pt/HT	80	6	87	0	<1	>99
10	Ag/HT	80	6	<1	-	-	-
11	Rh/HT	80	6	<1	-	-	-
12	Ru/HT	80	6	<1	-	-	-
13	Cu/HT	80	6	<1	-	-	-

^a^ Reaction conditions: Catalyst (Au: 0.9 mol%), toluene (5 mL), 80 °C, 6 h; ^b^ Determined by GC using an internal standard technique.

**Scheme 8:**
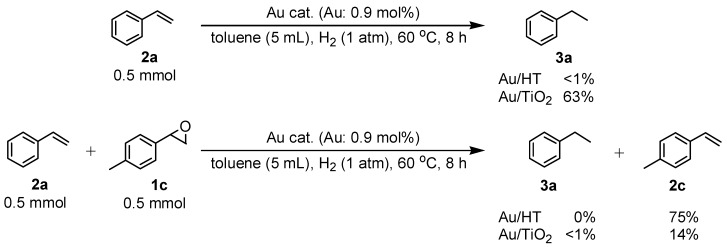
Hydrogenation of **2a** in the presence or absence of *p*-methylstyrene oxide.

Next, we conducted further studies on Au/HT and Au/TiO_2_ in the hydrogenation of **2a** in the presence or absence of *p*-methylstyrene oxide (**1c**) (Scheme 8). In the absence of **1c**, Au/TiO_2_ hydrogenated **2a** into **3a**. Surprisingly, Au/HT did not show any activity toward the hydrogenation of **2a**. Neither Au/HT nor Au/TiO_2_ hydrogenated **2a** in the presence of **1c**. These sharply contrasting results indicate that the hydrogen species generated on Au/HT are active for the deoxygenation of epoxides, but are completely inactive for the hydrogenation of C=C bonds. On the other hand, the high selectivity of Au/TiO_2_ for alkenes in the deoxygenation of epoxides (Table 5, Entry 6) is attributable to the preferential adsorption of epoxides over alkenes, which is a similar phenomenon to the previous report that the nitro group in 3-nitrostyrene was reduced by Au/TiO_2_ catalyst while retaining C=C bonds [49].

**Figure 5:**
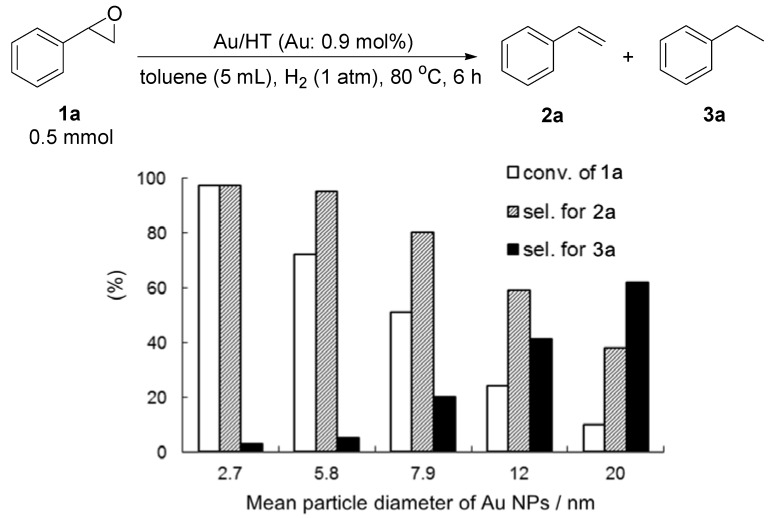
Size effect on the deoxygenation of **1a**.

Various Au/HTs with different mean diameters of Au NPs were tested for the deoxygenation of **1a** with H_2_ (Figure 5). Different sized Au/HTs could be prepared by varying the concentration of HAuCl_4_ solution [38]. Interestingly, larger Au NPs (>3 nm) showed lower catalytic activity and selectivity for the deoxygenation due to the hydrogenation of the C=C bond. The selectivity and yield of **2a** increased with decreasing mean diameter of supported Au NPs. From these results, it can be said that immobilizing small Au NPs (<3 nm) is the key to promoting the selective deoxygenation of epoxides to alkenes. The lower selectivity of larger Au NPs indicates that non-polar hydrogen species active for the hydrogenation of C=C bond were easily generated on the surface of larger Au NPs through the homolytic dissociation of H_2_. Au/HT with a mean diameter of 2.7 nm showed high catalytic activity and selectivity for the H_2_-driven deoxygenation of both aromatic and aliphatic epoxides to alkenes (Table 6). After the reaction, Au/HT could be reused with no loss of its catalytic efficiency (Entries 2 and 3).

**Table 6:** Deoxygenation of various epoxides using Au/HT ^a^. 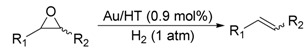

Entry	Substrate	Product	Time (h)	Conv. ^b^ (%)	Sel. ^b^(%)
1	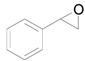	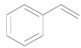	8	>99	>99
2 ^c^			8	97	>99
3 ^d^			8	97	>99
4	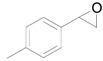	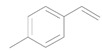	12	81	>99
5	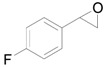	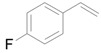	12	84	>99
6	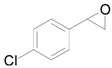	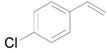	24	85	>99
7 ^e^	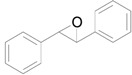	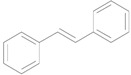	12	98	>99
8 ^e^	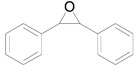	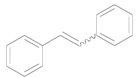	12	96	>99 (E/Z = 2/3)
9	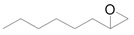	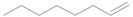	24	84	>99
10	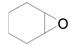	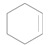	24	89	>99
11	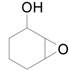	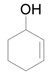	24	92	>99

^a^ Reaction conditions: Au/HT (0.1 g), toluene (5 mL), substrate (0.5 mmol), 60 °C; ^b^ Determined by GC or HPLC using an internal standard technique; ^c^ Reuse 1; ^d^ Reuse 2; ^e^ At 100 °C.

**Scheme 9:**
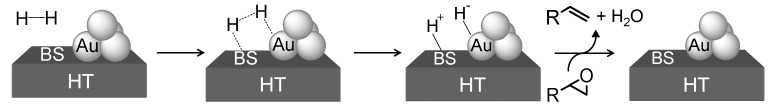
Heterolytic dissociation of H_2_ at the interface between Au NPs and basic sites (BS) of HT in the deoxygenation of epoxides.

Bearing in mind that basic ligands of transition-metal complexes promote heterolytic cleavage of H_2_ to give metal-hydride species, we propose a concerted effect between the basic sites of HT and Au NPs. Namely, heterolytic cleavage of H_2_ occurs to give [Au-H]^−^ and [HT-H]^+^ species at the interface between Au NPs and HT, which then react with an epoxide to afford an alkene and H_2_O (Scheme 9). The formation of [Au-H]^−^ and [HT-H]^+^ was confirmed by FT-IR studies. When Au/HT was treated with 50 mbar of H_2_, IR bands attributed to [Au-H]^−^ and [HT-H]^+^ appeared around 1,748 cm^−1^ and 3,200 cm^−1^, respectively. The exclusive formation of the heterolytically cleaved hydrogen species of Au-hydride and H^+^ on HT would selectively deoxygenate epoxides to alkenes.

## 3. Experimental

### 3.1. General

All organic reagents were purified before use. The products were identified by GC, HPLC and NMR analysis. Retention times (GC or HPLC) and chemical shifts (^1^H and ^13^C-NMR) of the products were in agreement with those of authentic samples (commercially available). HAuCl_4_·H_2_O was obtained from N. E. CHEMCAT. Co., Ltd. (Tokyo, Japan). MgO (GR for analysis) was purchased from Merck Chemicals Japan Co., Ltd. (Tokyo, Japan). Al_2_O_3_ (JRC-ALO-3), SiO_2_ (JRC-SIO-6) and TiO_2_ (JRC-TIO-4) were supplied by the Catalysis Society of Japan (Tokyo, Japan). Inductively coupled plasma measurements were performed by SII Nano Technology SPS7800. ^1^H and ^13^C-NMR spectra were recorded on JEOL JNM-AL400 and JNM-GSX270 spectrometers, respectively. GC (Shimadzu GC-2014) analysis was carried out with a KOCL-3000T and column. High-performance liquid chromatography (HPLC) was performed on a Shimadzu LC-10ADvp: STR ODS-IV. Atomic force microscopy (AFM) and transmission electron microscopy (TEM) micrographs were obtained with a Shimadzu SPM-9700 and Hitachi HF-2000 type microscope, respectively. Au L-edge X-ray absorption spectra were collected in the quick mode and recorded at room temperature in transmission mode at the facilities installed on the BL-01B1 line attached with a Si (311) monochromator at SPring-8, Japan Atomic Energy Research Institute (JASRI), Harima, Japan. Data analysis was performed using the REX 2000 program, ver. 2.5.7 (Rigaku). Fourier transformation (FT) of k_3_-weighted extended X-ray absorption fine structure (EXAFS) data was performed to obtain the radial structural function. FT-IR data were collected in a JASCO FT-IR 410 spectrometer equipped with a MCT detector. Self-supporting pellets were prepared from the sample powders and treated directly in the IR cell allowing thermal treatments under a controlled atmosphere.

### 3.2. Preparation of Au/HT

HT was prepared by the previously reported method [40]. Au/HT was synthesized as follows: HT (1.0 g) was added to 50 mL of an aqueous HAuCl_4_ solution (2 mM). After stirring for 2 min, 0.09 mL of aqueous NH_3_ solution (10%) was added. The mixture was further stirred at room temperature for 12 h. The obtained slurry was filtered and washed with deionized water, and dried in vacuo to afford HT-supported Au(III) species [Au(III)/HT] as a pale yellow powder. Au(III)/HT (0.9 g) was subsequently stirred in 50 mL of KBH_4_ solution (18 mM) under Ar atmosphere at room temperature for 1 h. The solid was filtered and washed with deionized water to give Au/HT as a reddish purple powder.

## 4. Conclusions

We discovered that Au/HT catalyzed the highly efficient deoxygenation of epoxides to the corresponding alkenes using various reductants. The obtained TON in the Au/HT-alcohol system was three orders of magnitude greater than that of previous reports. An alternative catalytic deoxygenation system was developed using CO/H_2_O as a reductant. The Au/HT-CO/H_2_O system could promote the deoxygenation of epoxides under mild reaction conditions (water, at room temperature, under 1 atm of CO). Under the water-gas shift reaction conditions, IR experiments revealed the *in situ* generation of the Au-hydride species. Finally, Au/HT realized an ideal green deoxygenation of epoxides using H_2_ as a reductant with water as the sole by-product. During these deoxygenations, no leaching of Au NPs from Au/HT to the reaction mixture occurred and Au/HT could be reused with retention of its high catalytic activity and selectivity. It is notable that the non-polar C=C bonds of products remained intact during these deoxygenations. The key to the above successful deoxygenation is the in situ generated Au-hydride and H^+^ species obtained through the concerted effect of the interface between Au NPs and basic sites of HT. We believe that this Au NP catalysis can be applied to other chemoselective reductions.
